# In-vitro cell treatment with focused shockwaves—influence of the experimental setup on the sound field and biological reaction

**DOI:** 10.1186/s40349-016-0053-z

**Published:** 2016-03-29

**Authors:** Kristin Dietz-Laursonn, Rainer Beckmann, Siegfried Ginter, Klaus Radermacher, Matías de la Fuente

**Affiliations:** Chair of medical engineering, RWTH Aachen University, Pauwelsstraße 20, Aachen, 52074 Germany; Department of Anatomy and Cell Biology, RWTH Aachen University, Wendlingweg 2, Aachen, 52074 Germany; Richard Wolf GmbH, Pforzheimer Straße 32, Knittlingen, 75438 Germany

**Keywords:** Shockwave, Therapy, In vitro, Experimental setup, Shockwave parameters, ESWT, Review

## Abstract

**Background:**

To improve understanding of shockwave therapy mechanisms, in vitro experiments are conducted and the correlation between cell reaction and shockwave parameters like the maximum pressure or energy density is studied. If the shockwave is not measured in the experimental setup used, it is usually assumed that the device’s shockwave parameters (=manufacturer’s free field measurements) are valid. But this applies only for in vitro setups which do not modify the shockwave, e.g., by reflection or refraction. We hypothesize that most setups used for in vitro shockwave experiments described in the literature influence the sound field significantly so that correlations between the physical parameters and the biological reaction are not valid.

**Methods:**

To reveal the components of common shockwave in vitro setups which mainly influence the sound field, 32 publications with 37 setups used for focused shockwave experiments were reviewed and evaluated regarding cavitation, cell container material, focal sound field size relative to cell model size, and distance between treated cells and air. For further evaluation of the severity of those influences, experiments and calculations were conducted.

**Results:**

In 37 setups, 17 different combinations of coupling, cell container, and cell model are described. The setup used mainly is a transducer coupled via water to a tube filled with a cell suspension. As changes of the shockwaves’ maximum pressure of 11 % can already induce changes of the biological reaction, the sound field and biological reactions are mainly disturbed by use of standard cell containers, use of coupling gel, air within the 5 MPa focal zone, and cell model sizes which are bigger than half the −6 dB focal dimensions.

**Conclusions:**

Until now, correct and sufficient information about the shockwave influencing cells in vitro is only provided in 1 of 32 publications. Based on these findings, guidelines for improved in vitro setups are proposed which help minimize the influence of the setup on the sound field.

**Electronic supplementary material:**

The online version of this article (doi:10.1186/s40349-016-0053-z) contains supplementary material, which is available to authorized users.

## Background

Shockwaves in medicine are mainly known for the extracorporeal comminution of stones, especially in the urinary tract. Besides their destructive character, shockwaves can also induce therapeutic effects in various tissues. This application is utilized in clinics worldwide especially for the treatment of various musculoskeletal disorders, for example, plantar fasciitis [1], nonunions [2], or calcific tendinitis of the shoulder [3]. Furthermore, the acoustic waves are also used therapeutically in several other medical fields like cardiology [4], dermatology [5, 6], and veterinary medicine [7]. However, despite its extensive use, there is still limited evidence concerning the therapeutic effects of shockwaves as there is only a limited number of high-quality clinical trials for some indications [8, 9]. Additionally, the underlying biological mechanisms of shockwaves on cells are unknown as well as the link to the inducing sound field. Therefore, the shockwave parameters required to induce a specific biological reaction are still unidentified and the development of standardized and optimized therapy concepts independent from the hardware is not yet possible [10–12].

In order to improve understanding of the tissue regenerating mechanisms induced by shockwave therapy, basic research is conducted in controlled laboratory environments using in vitro experiments. These studies show cell damage [13] on the one hand but also an increased expression of different growth factors associated with regeneration processes [14, 15]. The effects of shockwaves on cells and tissue seem to depend mainly on the number of pulses and the energy flux density [15, 16]. According to Kusnierczak et al. and Gerdesmeyer et al., the biological mechanisms of shockwaves are based on a threshold of the number of shocks and energy density which have to be exceeded to induce the biological reaction [16, 17].

**Fig. 1:**
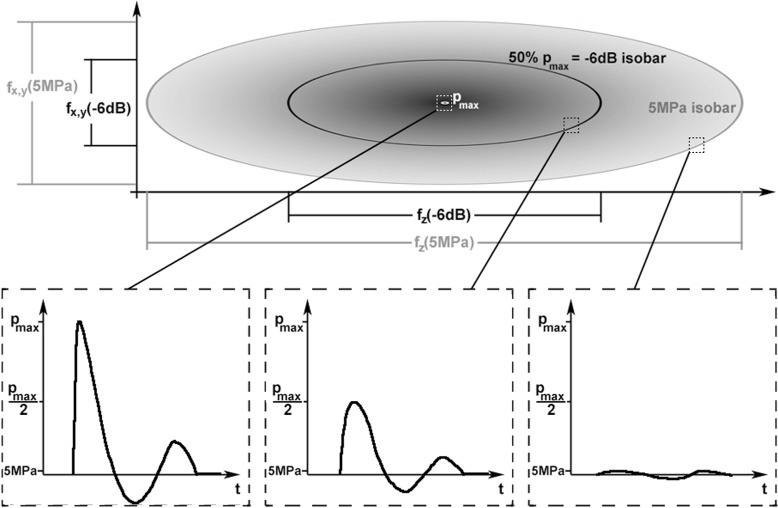
Schematic sound field (spatial distribution of maximum pressures) with definition of −6 dB and 5 MPa focal sizes according to IEC 61846 [18] and schematic pressure-time histories at three different positions of the sound field (focus, −6 dB isobar, 5 MPa isobar)

The energy (flux) density or “derived pulse-intensity integral” is one of the shockwave parameters defining the shockwave devices’ characteristic spatial and temporal pressure distribution. All shockwave parameters are measured by the manufacturers according to standard IEC 61846 [18] in a water bath which does not influence the sound field (free/undisturbed field). The pressure-time history of the focused wave is highly dependent on the position within the sound field (Fig. 1). Therefore, the maximum pressure of the field (*p*_max_) reaches only a very limited region while the local maximum pressures in the surrounding region decrease with greater distance from the focal point. According to the aforementioned standard, two definitions of the focal size are used: (1) the length and width of the sound field exceeding 5 MPa and (2) the −6 dB zone which equals the dimensions of the sound field reaching half the maximum pressure. The energy density can be calculated from every pressure-time history, but the energy density parameter of the shockwave device (ED) is calculated only from the focal curve and is therefore only valid at the focal point.

Over the years, different theories have been proposed on how shockwave therapy triggers the biological mechanisms. One possibility are microlesions occurring in the tissue after shockwave exposure which are assumed to stimulate tissue regeneration [19]. Despite the hypothesis by Johannes et al. stating that tissue repair is stimulated by enhancement of blood flow after shockwave therapy instead of direct cell stimulation [10], there are theories depending mainly on shockwave-induced mechanical deformation of the cells. This mechanical effect is a realistic assumption, as the rise time of a shockwave can be short enough to produce pressure gradients in a cell high enough to even fragment it [20]. Furthermore, there is shear strain due to the spatial pressure gradient of the shockwave which is amplified by subsequent pulses if the tissue does not relax in advance [21]. According to mechanotransduction studies, deformation of cells can lead to an activation of membrane ion channels and increased gene expression [22, 23]. Calculations indicate that this mechanobiologic effect can also occur during shockwave treatment. Based on thresholds of pore formation and membrane rupture by a real strain and tension, Lokhandwalla and Sturtevant predicted the formation of membrane pores and increased ion transport after shockwave application [24]. These results are in accordance with a study by Wang et al. who found a promotion of cell differentiation via membrane hyperpolarization after shockwave treatment [14].

Cavitation is another factor which may influence the biological reactions during shockwave treatment. Gas bubbles emerge from the medium due to the tensile forces of shockwaves, which is one reason why cavitation increases near material interfaces which invert the reflected shockwave (e.g., water-air) [25]. Apart from the pressure amplitude, also the temporal profile of the wave influences the occurrence of cavitation significantly [26, 27]. During implosion of the cavitation bubbles, secondary shockwaves and microjets are emitted leading for example to increased molecule transfection or cell destruction in nearby cells [20, 28, 29]. The large influence of cavitation on shockwave treatment results in vitro is also supported by studies showing higher cell damage of cells in the suspension in contrast to cells embedded in gel, where cavitation is suppressed [13, 30]. But while cavitation seems to be an important mainly destructive factor for applications near liquids like inside the kidney or blood vessels [27, 31], there is little information about shockwave-induced cavitation in more solid tissues [12, 32]. As there is little fluid in the space between cells in biological tissue, cavitational bubble growth might be restricted and its effects might differ from the condition in vivo [32]. Furthermore, the gas content of the fluids in vivo might differ from the one of the cell medium in vitro, thereby leading to varied cavitation effects [32]. The importance of cavitation for shockwave therapy in vivo is therefore still unknown.

As the latest theories concerning effective mechanisms of shockwave therapy include mainly mechanical effects, it is reasonable to expect that different sound fields lead to different biological reactions. This hypothesis is supported on the one hand by studies investigating the effects of direct sound field changes by using different driving voltages of the shockwave device. Results by Dongen et al. who treated a suspension of human prostate carcinoma cells showed differing cell survivals associated with the application of different shockwave fields (driving voltages 14.5 vs. 18.1 kV) [33]. And Maier et al. discovered a change in tensile strength of gastrocnemius tendons of turkeys following shockwave treatment with 25 kV (energy density of 1.2 mJ/mm^2^) in contrast to no measurable reaction at 15 kV (0.6 mJ/mm^2^) [34]. On the other hand, a sound field propagating towards a cell culture in vitro can be changed by the experimental setup due to physical effects like reflection, diffraction, or the generation of secondary shockwaves by cavitation [12, 35, 36]. The influence of the setup on the biological reaction has been demonstrated for example by Cleveland et al. in whose experiments the cell injury was significantly altered by a slight change of the shockwave field caused by different curvatures of the cell tubes [35]. Furthermore, experiments by Dongen et al., Steinbach et al., and Laudone et al. revealed a dependence of the cell death on air trapped inside cell tubes [33, 37, 38]. These examples show clearly that study outcomes can be strongly influenced by the experimental setup. For this reason, generally, no valid conclusions can be drawn by correlating the observed biological effects to the shockwave parameters measured by the manufacturer in the free field. Instead, the actual sound field reaching the cells in vitro has to be specified as it is the physical field inducing the biological reaction. This is a fundamental problem also in other closely related research fields concerned with cell exposure to acoustic fields [32]. To overcome this issue, guidelines are proposed, for example, which information about the acoustic source or experimental setup should be specified in articles concerning ultrasound exposure to cells [39]. The best possibility to obtain the sound field reaching the cells is the direct measurement with a fiber optic hydrophone. But the measurement equipment is quite expensive, the measurements are complicated and most researchers in this scientific field are clinicians with mainly clinical obligations and therefore limited time. For these reasons, direct measurements are usually not feasible. Another possibility is calculating the sound field using computer simulations [40]. But for realistic results and nonlinear calculations, a valid initial signal and realistic material parameters have to be defined. This is not trivial and requires profound knowledge about computer simulations and sound field measurements to verify the validity of the computational model. Hence, the easiest way to define the sound field acting on the cells is assuming that the manufacturers’ sound field parameters are valid in the vicinity of the cells. Thus, the in vitro setup cannot be allowed to influence the sound field significantly compared to the undisturbed free field measurements of the manufacturers.

In this paper, literature is reviewed and the most common in vitro setups used for shockwave experiments are identified and evaluated with special focus on their influence on the sound field. Based on this, guidelines for the improvement of shockwave in vitro setups are proposed which help to reduce the influence on the sound field. This opens up the possibility for valid correlations of the device’s shockwave parameters (measured by the manufacturer) with the biological reactions in vitro.

## Methods

### Literature review

Literature databases were searched for publications concerning in-vitro studies with focused shockwaves. Each “Methods” section was scanned for information about the experimental setup and shockwave parameters. If possible, missing information concerning the shockwave devices (e.g., principle of shockwave generation, focal dimensions) or the material of the cell container was obtained from manufacturer information or other publications. Care was taken to ensure that multiple publications of the same group in which equal experimental setups and procedures are described were included only once for evaluation (e.g., [13, 41] and [42–44]). All in all, 32 publications from 1988 to 2014 with 37 setups were considered. In order to characterize the setups used for in vitro shockwave experiments, the given information was evaluated with respect to the transducer technology, the cell container, the coupling medium between the transducer and cell container, and the cell or tissue model applied (Table 1). Furthermore, the features of the setups mainly influencing the sound field and the biological reactions were identified and evaluated. This included in particular:

1. Possibility of cavitation in the vicinity of the cells, as cavitation may lead to cell death and secondary shockwaves.

**Table 1:** Experimental setups used in published in-vitro shockwave experiments

Reference		SW	Coupling	Cell container		Cell model
Randazzo et al. 1988	[48]	eh	water	tube	PP	susp
Laudone et al. 1989	[38]	eh	water	tube	PP	susp
Gambihler et al. 1990	[56]	eh	water	tube	PP	susp
Cleveland et al. 1997	[35]	eh	water	tube	PP	susp
Delius and Adams 1999	[50]	eh	water	tube	PP	susp
Martini et al. 2003	[57]	eh	water	tube	PP	susp
Ueberle et al. 2002	[29]	eh	water	tube	PVC	susp
Hausdorf et al. 2010	[15]	eh	water	tube	?	susp
Braeuner et al. 1989	[30]	eh	water	pipette	PP	pel
Braeuner et al. 1989	[30]	eh	water	pipette	PP	gel
Strohmaier et al. 1990	[49]	eh	water	custom	silicone	susp
Maier et al. 2001	[34]	eh	water			organ
Wang et al. 2001	[14]	eh	cgel	tube	PS	susp
Yu et al. 2004	[53]	eh	cgel	tube	PS	susp
Zhang et al. 2014	[55]	eh	cgel	tube	?	susp
Neumann 2012	[70]	eh	cgel,mem,water	flask	PE	adh
Holfeld et al. 2014	[71]	eh	cgel,mem,water	flask	PS	adh
Ueberle et al. 2002	[29]	p	water	tube	PVC	susp
Moosavi-Nejad et al. 2006	[72]	p	water	custom	PE, glass	adh
Renz and Rupp 2009	[45]	p	cgel	tube	?	susp
Frairia et al. 2003	[42]	p	cgel	tube	PP	pel
Renz and Rupp 2009	[45]	p	cgel	tube	?	gel
Neumann 2012	[70]	p	cgel,mem,water	flask	PE	adh
Becker et al. 2014	[47]	p	med			organ
Dongen et al. 1989	[33]	em	water	tube	PP	susp
Yu et al. 1991	[51]	em	water	tube	PP	susp
Steinbach et al. 1992	[37]	em	water	tube	PP	susp
Steinbach et al. 1992	[37]	em	water	tube	PE	pel
Chao et al. 2008	[54]	em	water	tube	PS	susp
Hofmann et al. 2008	[58]	em	cgel	tube	PP	susp
Nurzynska et al. 2007	[46]	em	cgel	plate/dish	?	adh
Dorotka et al. 2003	[59]	em	cgel	well-plate	?	susp
Johannes et al. 1994	[10]	em	cgel,mem,water	tube	PP	susp
Oosterhof et al. 1989	[41]	em	cgel,mem,water	tube	PE	susp
Kusnierczak et al. 2000	[16]	em	cgel,mem,water	tube	?	adh
Gollwitzer et al. 2004	[52]	em	cgel,mem,water	pipette	PE	susp
Suhr et al. 2013	[73]	em	cgel,mem,med	plate/dish	?	adh

*SW* shockwave technology, *eh* electrohydraulic, *em* electromagnetic, *p* piezoelectric, *cgel* coupling gel, *mem* membrane, *med* cell culture medium, *custom* custom made, *PP* polypropylene, *PE* polyethylene, *PS* polystyrene, *PVC* polyvinyl chloride, *susp* suspension, *adh* adherent, *pel* pellet/sediment, *gel* embedded in gel, *organ* organ or tissue sample
2. The material of the cell container because the sound field inside it can be modified by reflection, diffraction, refraction, and absorption.
3. The size of the focal sound field in relation to the size of the cell model, as the treatment of a cell culture with a spatially highly inhomogeneous sound field might lead to an inhomogeneous cell reaction.
4. The distance between the treated cells and air because the sound wave is almost completely reflected and inverted at the air interface, thereby influencing the sound field reaching the cells.

In order to assess the influence of items 3 and 4 on the sound field, calculations and experiments were performed in addition to the literature review. The methods used are explained below.

### Relation between the −6 dB focal zone and cell model size

**Fig. 2:**
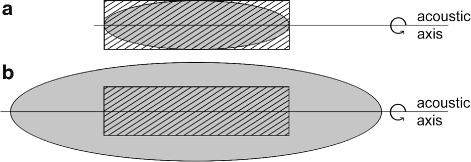
Two-dimensional view of the tube size (*shaded*) in relation to the −6 dB sound field size (*gray*). **a** Same sizes. **b** Sound field twice as big as the tube size

For focused shockwave transducers, the maximum acoustic pressure reaches only a very limited region (Fig. 1). If this region is smaller than the cell model, different cells are affected by significantly different temporal pressure distributions. In this case, a correlation of the shockwave parameters with the observed cell reactions is only valid if both are evaluated spatially. Linking the focal shockwave parameters with the mean cell reaction however leads to wrong conclusions. To define the maximum cell model size which ensures equal shockwave treatment of all cells, the percentage of cells treated with equal maximum pressures was calculated for different sound fields. The cell model was assumed to be cylindrical (e.g., suspension inside flat bottom tube) with length *L* of 45 mm and a radius *R* of 6 mm. These dimensions were used because many suspensions used for in vitro shockwave experiments are inside tubes of approximately that size (e.g., [33, 45]). The volume of the cylinder was calculated using *V*_cyl_=*π*·*R*^2^·*L*. The focal sound field was assumed to be an ellipsoid, and the focus was considered to be in the center of the cell tube with the cell model. The volume of the sound field can thus be calculated using $V_{\text {el}}=\frac {4}{3}\pi \cdot r^{2} \cdot \left (\frac {l}{2}\right)^{2}$. The dimensions *r* and *l* depend on the −6 dB sound field. In the first case, the −6 dB sound field was assumed to have the same dimensions as the cell model (Fig. 2a). In the second case, the sound field size was chosen twice as big (Fig. 2b). For calculations of the percentage number of cells treated with pressure-time distributions between 100 % and lower than 50 % of the maximum pressure (within sections of 10 %), the pressure distribution along all main axes was assumed to be a Gaussian curve (see [11]). To get the amount of cells treated with a certain percentage of the maximum pressure, the corresponding ellipsoid volume was divided by the cylinder volume.

### Influence of water-air interfaces on the sound field

During shockwave treatment, air is very often in direct vicinity to the cells and the focal region of the sound field. Due to sound reflection of almost 100 % at water-air interfaces, the forces on the cells can change significantly, depending on the distance between the cells in the focal region and the material interface. Furthermore, the wave is inverted during reflection, leading to high strains and possibly additional cavitation. To evaluate the influence of the distance of those interfaces on the forces acting on a cell model, a pellet (diameter 2 mm, weight 4 mg) made of modelling dough was placed in a tube (1.5 ml, Sarstedt Safe Seal, 72.706), filled with water, and treated with shockwaves. To simulate the cell culture medium used for real cell experiments, nondegassed tap water was used inside the cell tube and the distance from the submerged pellet to the air interface on the acoustic axis was varied from 1 mm to complete filling (35 mm). The treatment was conducted inside a measurement tank filled with degassed and deionized water. The pellet inside the tube was positioned in the focus of an F10G4 shockwave transducer (Richard Wolf GmbH) and a pulse repetition frequency of 1 Hz and intensity level of the Piezoderm (Richard Wolf GmbH) of 20 (corresponds to 7 kV driving voltage, *p*_max_= 77.7 MPa, *p*_min_=−18.7 MPa, 5 MPa focal zone *f*_*z*,5 MPa_= 4.4 cm in the free field) were applied for approximately 1 min. Videos of the pellet movement during treatment (Additional file 1: Video S1 and Additional file 2: Video S2) were analyzed with respect to the pellets’ maximum acceleration using the open source software VianaNET (version 5.0.3). Standard deviations were obtained by repetition of the experiment as well as repetition of the video analysis. The 0.05 significance level was calculated from the results with the completely filled tube using a *t*-test. The force on the pellet can be calculated by *F*=*m*·*a* with the acceleration *a* and the mass of the pellet *m*.

## Results

**Fig. 3:**
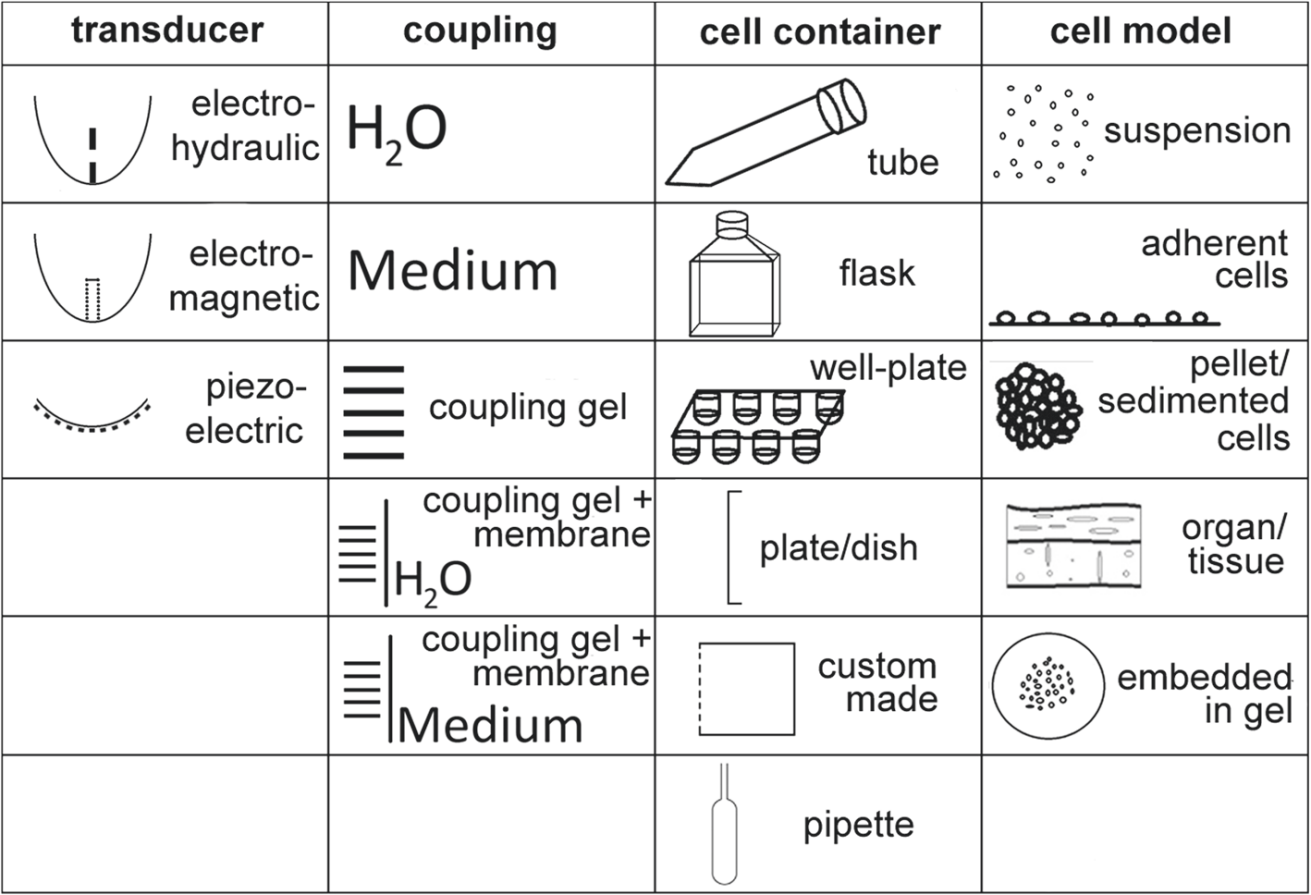
Possibilities for in vitro setups used in shockwave research

**Fig. 4:**
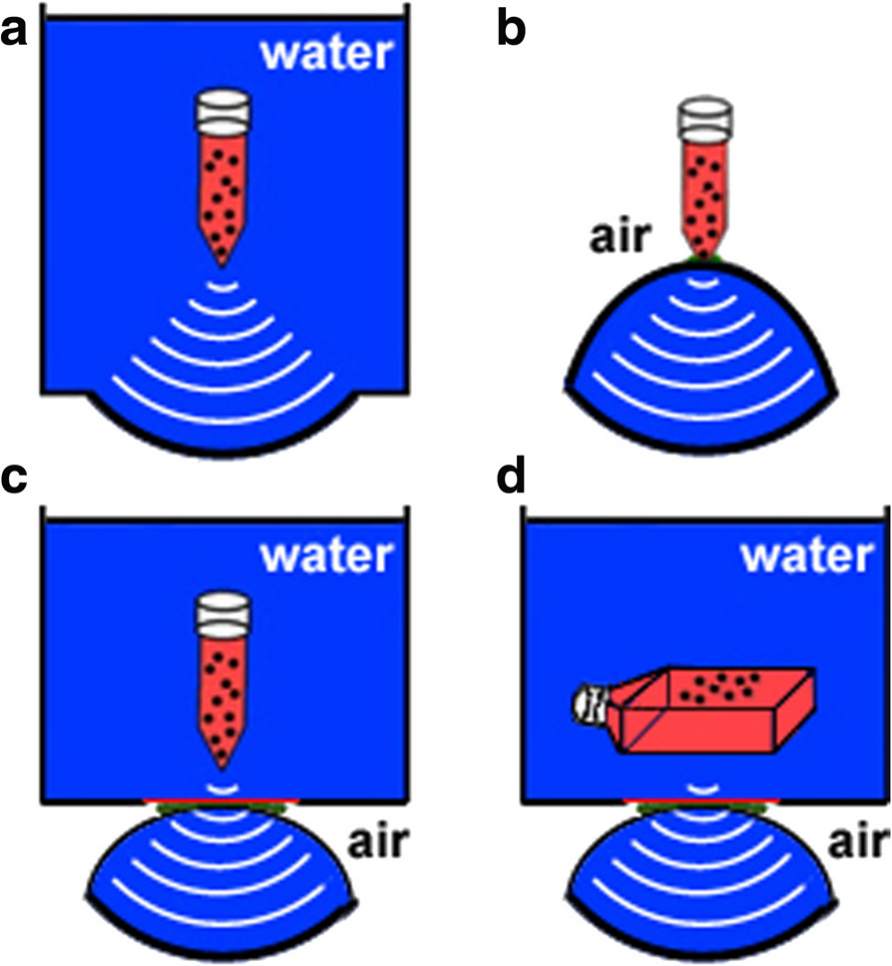
The mainly used setups for in vitro shockwave experiments: tube with single cell suspension in a water bath coupled to a shockwave transducer by water (**a**), tube with single cell suspension coupled to a shockwave transducer with coupling gel and surrounded by air (**b**), ultrasound gel coupling of the transducer to a water basin with a membrane, and either a tube with cell suspension (**c**) or a cell culture flask with adherent cells (**d**)

All reviewed in vitro setups for focused shockwave experiments published since 1988 can be categorized concerning the used shockwave transducer, the cell or tissue model, the cell container, and the medium used for acoustic coupling between the transducer and the cell container (Fig. 3). The results of the literature review of in vitro setups used for focused shockwave experiments are summarized in Table 1. In all 37 setups, 17 different combinations of coupling, cell container, and cell model are described. The setup used mainly is a transducer coupled via water to a tube filled with a cell suspension (13/37) (Fig. 4a). Other important setups are a transducer coupled directly via coupling gel to a tube filled with a cell suspension (5/37) (Fig. 4b) and ultrasound gel coupling of the transducer to a water tank with a membrane and either a tube or pipette with cell suspension (3/37) (Fig. 4c) or a cell culture flask with adherent cells (3/37) (Fig. 4d).

Besides the number of shockwaves applied and the repetition frequency, the shockwave parameters of the device specified in the publications are mainly the energy density (23/37) and the maximum pressure (16/37). These are usually either manufacturer information or cited from other publications often using a different setup (e.g., [46]). Direct measurements of the parameters in the setup and at the position of the cell model are only conducted in 5 of the 32 publications [33–35, 37, 41].

### Transducer

The technology available for shockwave transducers is either electrohydraulic, electromagnetic, or piezoelectric. But mainly electrohydraulic (46 %) and electromagnetic (35 %) shockwave generators are applied. For coupling of the shockwave to the target region, the reflector or piezoceramic surface of the shockwave transducer is either in direct contact with the coupling medium or water-filled cushions or gel pads are attached to the transducer surface. In those cases, the coupling medium is in contact with the cushion membrane.

### Coupling

In most cases (51 %), the sound coupling medium between the transducer and the cell container (or in a few cases directly tissue or an organ) is water with both the shockwave transducer and the cell container being submerged in a water tank (Fig. 4b). Instead of water, also, cell culture medium can be used to couple the transducer surface to the tissue as it was done by Becker et al. [47]. Other coupling methods need coupling gel (e.g., ultrasound gel) either for direct coupling of the transducers’ water cushion or gel pad to a cell container (9/37) or for the acoustic contact with a membrane followed by a basin with water or cell culture medium (8/37). In the second case, only the cell container is surrounded by fluid while the cushion of the transducer is surrounded by air except for the contact area to the membrane (Fig. 4c, d). Direct coupling implies that the transducer and the cell container are in direct contact via gel and both completely surrounded by air (Fig. 4b). Although both coupling gel groups each represent only about 24 % of all setups, those setups have gained importance in the last years, as water coupling has been used less. Taking a look at only the setups used since the year 2000 reveals almost equal application of all the three main setups (each 6-9/23) with direct coupling (Fig. 4b) being the mainly used setup.

### Cell model and container

In 23 of 37 setups, a cell suspension is applied as a cell model while adherent cells are utilized in fewer cases (7/37). Combined, all other models like pellets or sedimented cells, cells embedded in gel and whole organs or tissue samples are only used in a minority of 19 % of the published in vitro setups. The cell containers used are any of the standard cell containers available. These are in detail different tubes (test tubes, cryotubes, micro tubes, centrifuge tubes), cell culture flasks, well plates, plates or dishes (also cover slide glass), plastic Pasteur pipettes, or custom made. The cell container is always at least partly filled with the cell model and cell culture medium. The cells are usually inside a tube made of either polypropylene (PP) (38 %), polyethylene (PE) (16 %), or polystyrene (PS) (11 %) which are common cell container materials. For 22 % of the setups, the tube material is neither specified directly nor can it be deduced from other information.

**Fig. 5:**
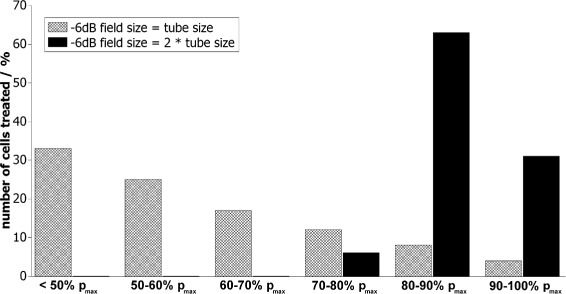
Histogram of the maximum pressure (relative to the maximum focal pressure) reaching a cell suspension (length, 45 mm; radius, 6 mm) in case of two different −6 dB sound fields

In case of suspensions, the size of the cell model relative to the sound field is mostly either not sufficiently specified (57 %) [15, 29, 35, 38, 41, 48–55] or in the order of magnitude of the −6 dB focal zone or even bigger (30 %) [10, 14, 37, 45, 56–58]. Assuming that the material and geometry of the tube do not affect the sound field, calculations show (Fig. 5) that only 4 % of the cells inside a tube with the same dimensions as the −6 dB focal lengths (geometry: Fig. 2a) are treated with nearly the maximum pressure (90–100 % *p*_max_). But more than 50 % of all cells are reached by less than 60 % of the maximum pressure. This is due to the spatial pressure dependence of the sound field (Fig. 1). For wider sound fields, this relation changes, so that with a −6 dB sound field of twice the tube size (geometry: Fig. 2b), all cells are almost equally treated with more than 80 % of the maximum pressure. However, even in that case, not all of the cells are treated with the maximum pressure of the shockwave field (measured by the manufacturer), but on average with only 87 % of it.

### Vicinity of air

In many setups, air is very close to the focal region and the cell model, for example:

- when air is trapped in a cell tube with a pellet or cell suspension (e.g., [38])
- if a 96-well plate is used [59]
- if the transducer is directly coupled to a cell tube via ultrasound gel (e.g., [42])

**Fig. 6:**
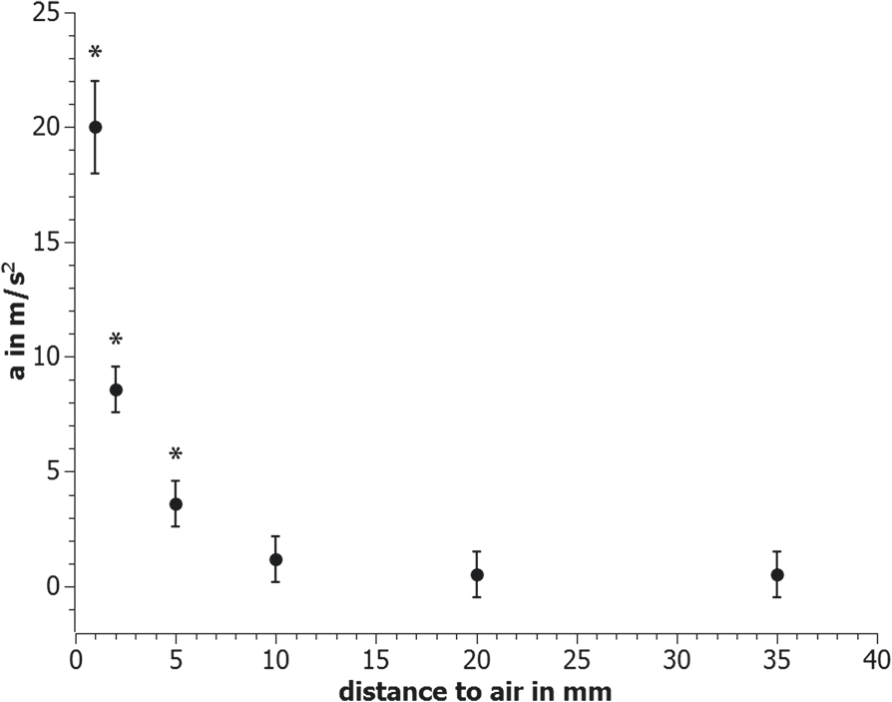
Shockwave-induced maximum acceleration of the modelling dough pellet in the dependence of the pellet-air distance. Significant increases (significance level 0.05) compared to the completely filled tube are marked (*asterisk*)

Especially in the first two cases where the air is on the acoustic axis directly behind the focal region, reflections at the water-air interface significantly change the sound field and thus the forces on the cells. This is demonstrated by our experimental results of the pellet acceleration analysis. They show a significant increase of the acceleration (and thereby the force) for pellet-air distances of ≤5 mm compared to complete tube filling ($\widehat {=}$ 35 mm) without air pockets (Fig. 6). Changing the distance of the cell model to air from complete filling to 1 mm increases the maximum forces considerably by a factor of 40.

### Cavitation

In order to avoid cavitation, the water used for shockwave in vitro setups is in 16 of 37 setups explicitly stated to be degassed. But the resulting gas content is only mentioned in two cases [50, 56], so that the quality of degassing can only be assessed in those two cases. Moreover, none of the publications states the gas content or a degassing process of the cell culture medium which usually directly surrounds the cell model. Cavitation can hence only be ruled out in one setup described by Renz and Rupp where the cells are embedded in alginate gel and ultrasonography jelly is used to couple the transducer to the cell container [45].

## Discussion

One main problem for the credibility of the effectiveness of shockwave therapy is that the underlying effective mechanisms are still unclear. This means, it is unknown which of the physical characteristics of the shockwave are responsible for which reaction on the cellular level. Thus, a main objective of shockwave research is correlating the physical properties of the shockwave with the biological reactions of the cells or tissues. The prerequisite to compare different study outcomes and thereby investigate this correlation is the measurement of the shockwave-induced biological reactions on the one hand. On the other hand, there is also the need to quantify the sound field reaching the cells and inducing these cellular responses. But while the measurement of the biological reactions is a standard in shockwave research, the quantification of the sound field inducing these reactions is rare.

There are several possibilities to obtain the sound field reaching the cells. Direct measurement in the in vitro setup and computer simulations are two of them. But they are both complicated and require special measurement and simulation equipment as well as theoretical knowledge of simulation techniques and shockwave measurement experience. As most of the shockwave researchers are physicians with mainly clinical obligations, these possibilities are usually not feasible. Therefore, the best choice is using an in vitro setup which does not influence the sound field significantly for example by reflection, diffraction, or secondary shockwaves. In this case, the sound field parameters in vitro would be equal to the manufacturers’ free field measurements. In order to distinguish between insignificant and significant sound field variations, the influence of sound field variations on the biological reaction was reviewed and a limit for significant sound field variations can be defined as discussed below.

The experimental data collected by Dongen et al., Steinbach et al., and Maier et al. show different biological reactions after applying different energy densities (0.32 vs. 0.21 mJ/mm^2^ [37]) or maximum pressures (110.6 vs. 87.5 MPa [34]; approx. 27.5 vs. approx. 31 MPa [33]). From these data, we can conclude that a reduction of the maximum pressure reaching the cells of 11 % can already significantly change the in vitro cell reaction. Such a reduction of maximum pressure and energy can not only occur by changing the driving voltage of the shockwave transducer as it was done in the aforementioned studies but also by placing a sound reflecting material like a cell container in the acoustic path of the shockwave. If the materials lead to a reduction of the transmitted maximum pressure of approximately 11 % (i.e., 89 % of the generated shockwave pressure), the modification of the shockwave parameters relative to the free field may not be neglected as they could potentially influence the biological reaction. The transmitted pressure *p*_*t*_ behind a material interface (e.g., water-cell container) can be calculated from the incident pressure *p*_*i*_ (=generated shockwave pressure) by *p*_*t*_=*T*·*p*_*i*_. The acoustic transmission coefficient *T* is defined by the acoustic impedance *Z* of the materials in front of (1) and behind (2) the interface: $T = \frac {2Z_{2}}{Z_{1} + Z_{2}}$. In case of a cell container, the wave is transmitted through two material interfaces before reaching the cells—from water (*W*) into the cell container (*C*) and at the rear side of it back into water. The resulting directly transmitted wave through both interfaces can be calculated using $p_{t\_{\text {tot}}} = p_{i} \cdot T_{\text {tot}} = p_{i} \cdot T_{C} \cdot T_{W} = p_{i} \frac {4 \cdot Z_{W}\cdot Z_{C}}{(Z_{W}+Z_{C})^{2}}$. Apart from the direct wave, the transmitted wave is superimposed by a wave which is reflected inside the container material and partly transmitted at its rear side. However, this wave is shifted temporally in relation to the directly transmitted wave due to the extension of the traveled distance. In case of a cell container of about 1 mm material thickness, the temporal shift due to reflection in the materials PE, PVC, PP, and PS from Table 2 is about 0.8 *μ*s. As the shock front of the wave usually lasts only about 1 *μ*s, the time shift of the internally reflected wave is sufficiently high to have no influence on the maximum transmitted pressures. However, the overall shape of the transmitted pressure time curve can be significantly changed by this effect. Apart from reflections, the transmitted sound wave is attenuated by the cell container material. For cell containers of usually about 1 mm material thickness, this effect is negligible for most cell container materials. Taking a look at different cell container materials used (see Table 2) reveals a total direct pressure transmission through a thin slice of the cell container materials between 95 and 34 %. As discussed before, it is quite possible that a transmission of 89 % could already result in a different biological reaction compared to the one which would occur after treating the cells with the undisturbed shockwave field. Therefore, if a cell container made of PVC or glass is used, the correlation of the cell reaction with the physical parameters of the shockwave measured by the manufacturer in the free field will likely lead to wrong conclusions. In addition to the direct energy and pressure loss by reflection, also, a decreased effect of nonlinear propagation is expected behind the reflecting material, whereby the formation of the steep shock front is diminished [60]. This lead to even lower pressures reaching the cells inside a container than calculated above.

**Table 2:** Cell container materials and their resulting transmission coefficients *T*_tot_ for transmission of acoustic pressures through a thin slice of container material (index *C*) in water (index *W*)

Cell container material			*ρ* / kg m ^−3^	*v* / ms ^−1^	*Z* / 10 ^−6^ kg m ^−2^s	*T*_tot_ / %
Glass			2490	5840	14.54	34
PP	(polypropylene)	@ 25 °C	913	2650	2.42	94.5
PE	(polyethylene)	@ 25 °C	957	2430	2.33	95.3
PS	(polystyrene)	@ 25 °C	1052	2400	2.52	93.6
PVC	(polyvinyl chloride)	@ 25 °C	1386	2330	3.23	86.6
Water		@ 30 °C	996	1509	1.50	100

Material constants from [72, 74, 75]

The total transmission coefficients are calculated using $T_{\text {tot}} = \frac {4 \cdot Z_{W}\cdot Z_{C}}{(Z_{W}+Z_{C})^{2}}$ and acoustic impedance *Z*=*ρ*·*v* with density *ρ* and longitudinal sound velocity *v* of the material

According to those calculations, the cell container materials polypropylene, polyethylene, and polystyrene do not significantly disturb the sound field by reflection. But as Cleveland et al. have demonstrated, the geometry of a round bottom polypropylene tube can influence the sound field by refraction and thereby change the biological reactions of a cell suspension significantly [35]. As refraction does not occur with waves that impinge perpendicular to the interface, the relation between the curvature of the tube and the curvature of the sound field influences the aforementioned effect. This means that in the particular case of a round bottom tube with the same curvature as the sound field and exact positioning at the focal point, the effect will be eradicated. However, in general, the refraction effect can occur inside any standard cell container, thereby changing the cellular response to the sound field. The sound field parameters influencing the cells inside standard tubes can therefore only be assessed by either direct pressure measurements inside the tube, use of cell containers made of a material and with a geometry which do not influence the sound field significantly, or not using any cell container at all. Additionally, there is the possibility to calculate the sound field inside a tube. But in order to do this, some parameters of the sound field and the tube have to be known like the acoustic parameters of the tube material, its geometry, its position relative to the focus point of the sound field, the curvature of the transducer, and the sound field in front of the tube. As these calculations might become rather complicated and some of the parameters needed are usually not known, this possibility might only be applicable by few researchers.

If the size of the cell suspension relative to the sound field is specified in publications, it is usually in the order of magnitude of the −6 dB focal zone (Fig. 2a) or even bigger. This leads to a severely differing treatment of all cells reaching from 100 % to less than 50 % of the transducers maximum pressure (Fig. 5). While only 4 % of the cells are treated with 90 to 100 % of the maximum focal pressure, on average, only 59 % of *p*_max_ is applied to the whole cell culture. To investigate the correlation between the shockwave parameters and the cell response, the treatment of all cells with well-known sound field parameters has to be ensured. Therefore, a suspension with the dimensions of the −6 dB shockwave field is not a useful in vitro setup for shockwave research. Even if the −6 dB sound field is twice as big as the cell model (Fig. 2a), there is still a mean difference of about 13 % between the specified and the effective shockwave parameters. As discussed before, this difference might already result in varied biological reactions and therefore in a wrong correlation of the physical parameters and the biological reaction. Additionally, the cells can move in the suspension due to acoustic streaming [39], whereby the exposure to the sound field becomes even more unpredictable for each cell. On the other hand, if the cells are fixed at one position during the whole experiment (e.g., adherent, 3D model or embedded in gel), the biological reaction might be evaluated with respect to its position as done by Bräuner et al. [30]. If apart from that also the sound field is evaluated spatially, the relation between the −6 dB shockwave field and the cell model size does not represent any limitation to the quality of the in vitro setup. This theoretical evaluation of the cell number reached by shockwaves with different percentages of the maximum pressure is limited on the one hand by the assumed cylindrical geometry of the tube bottom which is usually either conic or round. Realistic geometries would lead to a smaller total size of the cell model and a restriction of the cell model in the tube bottom to the higher pressure region. This leads to a slight increase of the percentage of cells treated with the maximum pressure. On the other hand, the curvature of the tube bottom leads to additional focusing of the sound wave [35] which in turn narrows the sound field and leads to a decreased number of cells treated with the highest pressures. As both limitations have opposite influences on the sound field, they might cancel out so that the evaluation seems to be a good first approximation of the reality.

Apart from the cell container and the cell model dimension, also, other parts of the setup have a significant effect on the sound field. Especially, the distance of the cells in the focal region to air can change the biological response significantly. Dongen et al., Steinbach et al., and Laudone et al. [33, 37, 38] all found a significantly higher cell death in partially filled tubes containing air in contrast to completely filled tubes. This effect presumably arises because of the almost complete reflection and phase inversion of the sound wave at any water-air interface. In our experiments, focal region to air distances on the acoustic axis below 1 cm lead to significantly increased forces on a cell model at the focus (Fig. 6). For the transducer used, this corresponds to half the 5 MPa focal length. Different forces acting on cells during in vitro experiments can lead to a significant change of the biological reaction, like the aforementioned cell death rate. In order to ensure an insignificant influence of the air on the experimental results, we therefore recommend that no water-air interfaces should be present within the 5 MPa focal zone on the acoustic axis. But also, water-air interfaces which are not on the acoustic axis might influence the sound field. Those interfaces exist for example in the mostly used setup of the last 15 years in which direct coupling of the shockwave transducers’ water cushion or silicone pad to a cell tube is used (Fig. 4b). As the membrane of the cushion is surrounded by air except for the coupling site, all radial acoustic waves are inverted and reflected towards the cell tube. Usually, some parts of the shockwave propagate in radial direction. These are especially the primary wave of electrohydraulic transducers and the spherical elementary waves produced at any point of a shockwave transducer or reflector surface. Compared to the free field, a setup in which the transducers’ coupling cushion is surrounded by air therefore presumably leads to significantly influenced shockwave fields in the focal region. Apart from these reflection-induced pressure modifications, the use of coupling gel is generally problematic because air bubbles can be trapped in the gel. This leads to a significantly reduced transmission of the shockwave towards the target region. Depending on the coupling procedure, in particular breaking contact and recoupling, the percentage of the coupling area which is covered with air is highly variable resulting in significantly decreased and differing shockwave amplitudes [61]. The validity and reproducibility of shockwave in vitro setups using coupling gel is therefore doubtful.

During reflection at a water-air interface, there is also phase inversion. This means that the high maximum pressures of the shockwave turn into high tensile stresses which in turn increases the cavitation potential. Cavitation is a chaotic, not accurately reproducible, and difficult to predict phenomenon. The bubbles produced by the high tensile stresses can on the one hand attenuate the sound field because of reflection and absorption of the shockwaves by the bubbles [62, 63]. On the other hand, the bubbles implode forming a shockwave or microjets which might influence the cells. In in vitro experiments with shockwaves cavitation is usually held responsible for cell damage [30, 45, 56]. But while cavitation seems to be an important mainly destructive factor for applications in the vicinity of liquids like inside blood vessels or during lithotripsy inside the kidney [27, 31, 64], there is little information about shockwave-induced cavitation in more solid tissues [12]. It is thus questionable if cavitation is involved in the therapeutic effect of shockwaves in vivo. But cavitation is a disruptive factor for the reproducibility of laboratory experiments and can already be altered by the presence of even a small bubble in the cell medium [65]. Therefore, it should be avoided for in vitro shockwave experiments. This might either be done by applying only shockwaves with limited negative pressure amplitude or by degassing the medium in the sound path from the transducer to the cells as well as the medium directly surrounding the cells. Additionally, the existence of cavitation in vitro can be easily checked with a clinical ultrasound imaging system as cavitation is visible by ultrasound as dynamic white clouds with a temporal relation to the shockwaves [66].

To find a correlation between the physical properties of the shockwave and the induced cell reactions, the shockwave and the biological results both have to be reproducible and well defined. According to standard IEC 61846 [18], most shockwave parameters, especially the focal −6 dB area and the energy (flux) density (or pulse intensity integral) are defined from the focal pressure-time measurement or in relation to the focal maximum pressure. But as electrohydraulic shockwave sources generate highly varying shockwaves (in position and amplitude) with every pulse [67, 68], the exact measurement of the focal pressure time history is almost impossible. Therefore, the set of shockwave parameters cannot be defined correctly for electrohydraulic shockwave devices. As a result, those devices are not well suited for reproducible experiments aiming on correlating shockwave parameters to the biological reaction. Also, different sound field data from the literature might not be directly comparable, as the hydrophone measurement systems developed over time. While in older studies PVDF sensors were used which underestimate the negative pressure of the wave, newer studies usually use fiber optic sensors with which this problem does not occur [69].

The energy (flux) density (ED) of the free field is a shockwave parameter which is stated in most articles, and very often, it is used as a comparative parameter. But this parameter is not well suited to compare different devices and resulting biological effects. This is because the energy density value is only valid exactly at the measurement point (usually the focal point), which is a very small area of the sound field (approx. 0.008 mm^2^ for a fibre optic hydrophone). Additionally, this value does not contain any information about the spatial or temporal characteristics of the shockwave field. Hence, the energy density value can be equal for completely different sound fields (e.g., strong vs. weak focusing) and different focal pressure time histories (e.g., shockwave vs. continuous ultrasound wave). Therefore, ED is an inadequate shockwave parameter to compare shockwave studies.

Before useful shockwave parameters can be identified which enable a comparison of the devices based on their resulting biological reactions, a correlation of those parameters and the cell reaction has to be researched. But as discussed before, the sound field is significantly influenced by many of the setups usually used for in vitro shockwave studies.

### Are the sound field parameters provided by the reviewed publications valid in the vicinity of the treated cells?

One possibility to obtain the sound field reaching the cell model is using a setup which does not influence the sound field relative to the free field measurements in water. But the associated requirements discussed above are met by none of the 37 setups reviewed. Another option to get valid shockwave parameters is conducting reproducible measurements of the sound field in the setup used for the in vitro experiments at the position of the cell model. This has only been done by Dongen et al., Maier et al., Cleveland et al., Steinbach et al., and Oosterhof et al. [33–35, 37, 41]. But Maier et al. and Cleveland et al. [34, 35] use electrohydraulic shockwave generators. As their sound field is highly variable with every pulse, the shockwave parameters can only be determined on average. Therefore, the parameters are hardly comparable to the ones of electromagnetic or piezoelectric shockwave transducers. Additionally, the use of cell suspensions is problematic if they have the same dimension as the −6 dB focal size or unknown size relations as in the publications by Maier et al., Cleveland et al., Steinbach et al., and Oosterhof et al. [34, 35, 37, 41]. This is because the treatment parameters change significantly throughout the cell model, and the biological evaluation cannot be limited to individual sections.

The effective mechanisms of shockwaves cannot be concluded by reviewing the existing publications of in vitro shockwave experiments. Until now, only the article by Dongen et al. [33] seems to provide sufficient information about the sound field reaching the cell model and the resulting biological reaction so that a comparison of several studies and their results cannot be conducted. Therefore, new in vitro shockwave experiments are required which focus not only on the shockwave-induced biological reactions but also on providing valid information about the sound field reaching each cell during the experiment.

## Conclusions

Correct and sufficient information about the sound field reaching the cells is only provided in 1 of 32 publications. A comparison of experimental results and conclusions concerning probable effective parameters is therefore currently impossible. In order to allow for comparable in vitro studies with focused shockwaves, the sound field reaching the cells as well as the induced biological reaction have to be described. The best way to specify the acoustic field of the shockwave acting on cells during in vitro experiments is the direct measurement of the sound field in the setup and at the exact position of the cell model. If direct measurements cannot be conducted, the shockwave parameters provided by the manufacturer have to be used. But as those parameters are measured in a water bath and therefore represent the undisturbed free field of the shockwave transducer, the parameters are only valid for in-vitro experiments, if the experimental setup does not influence the acoustic field significantly. To achieve this, the below guidelines should be followed:

- Use cell model dimensions smaller than half the −6 dB focal dimensions of the sound field or immobile cells evaluated spatially
- Avoid air within the 5 MPa focal zone
- Avoid standard cell containers $\rightarrow $ use either no cell container or materials and geometries which do not influence the sound field
- Avoid cavitation: use either low energy levels or media with a high enough cavitation threshold
- Avoid coupling gels
- Avoid electrohydraulic transducers

Apart from following these guidelines for standardized in vitro setups, an optimization of the in vitro experiments can best be achieved by interdisciplinary collaboration of engineers, physicists, biologists, and physicians.

## Additional files

**Additional file 1:** Video 1. Shockwave treatment of the pellet model with 2 mm distance from the focal point to air. (MOV 1587 kb)

**Additional file 2:** Video 2. Shockwave treatment of the pellet model with 35 mm distance from the focal point to air (completely filled). (MOV 5130 kb)
